# The Hospital. Nursing Section

**Published:** 1904-12-31

**Authors:** 


### The Hospital.

### Nursing Section.

Contributions for this Section of "The Hospital" should be addressed to the Editor, "The Hospital," Nursing Section, 28 & 29 Southampton Street, Strand, London, W.C.

No. 953.—Vol. XXXVII. SATURDAY, DECEMBER 31, 1904.

### Notes on News from the Nursing World.

### MINISTERING TO THE RUSSIAN WOUNDED.

In another column will be found a graphic description of the sufferings of some of the victims of the war between Russia and Japan, written by a sister engaged in a Russian-Dutch field hospital. The regret with which it will naturally be read will be tempered by satisfaction that the poor fellows appear to have been nursed in the true nursing spirit. In the Far East, as in South Africa, the soldier in his last agony turns to the sister in attendance, and shows his appreciation of her kindly offices. It is so all the world over, without respect to nationality, wherever the nurse has not mistaken her vocation. But this is the one essential part of her duty for which no amount of training can fit her.

### MALE AND FEMALE MENTAL NURSES.

The medical superintendent of Stirling District Asylum warmly protests against any attempt to depreciate a particular class of asylum workers, and we agree with him that "the interests of male nurses are best advanced by bringing forward the many strong claims they have for support." Nothing is more calculated to injure these interests than attempts to glorify their qualifications at the expense of nurses of the other sex. But we do not think that Dr. Robertson need be concerned about the recommendation of Dr. Urquhart that women should be excluded from all male wards in asylums. Dr. Urquhart is a high authority on mental nursing, which he has shown he is very anxious to improve. But it is too late in the day to urge that women should be prevented from taking positions which they have already occupied with satisfactory results.

### WHAT IS A TEMPORARY NURSE?

The Local Government Board having refused to sanction the appointment of a young woman as a staff nurse at the Blackwall branch of the Poplar and Stepney Sick Asylum because she is under 21, the managers decided at their last meeting not to fill up the vacancy, but to place the young woman on the temporary staff for twelve months at a salary of £25 per annum with allowances. It appears to us that if the managers conform to regulations of the Local Government Board in the letter they disobey them in the spirit.

### ST. BARTHOLOMEW'S HOSPITAL.

The engagement of a probationer at St. Bartholomew's Hospital to a gallant admiral is described in the daily press as the "romance of a nurse"; but although the probationer, who has been interviewed respecting her adventure, speaks of "dear old Barts," she has only been in the hospital for three months. We learn with more pleasure the fact that the members of the League of St. Bartholomew's Hospital Nurses, with the nurses at present training in the institution, have collected £1,500 towards the rebuilding fund. This money, and another donation at a later date, will be devoted to the erection of a nurses' home.

### GUARDIANS AND DISTRICT FEES.

The Petworth Guardians may be concerned to be primarily regarded as vigilant custodians of the ratepayers' money, but we do not think that they would have been accused of extravagance if they had defrayed the entire expense of the nurse attached to the Sussex County Nursing Association who recently attended a patient in the workhouse and practically saved her life. At the suggestion of the chairman, a cheque for two guineas has been sent to the Association; but, as the nurse was in attendance for a month, at an actual cost of 17s. 6d. per week, the amount is scarcely liberal enough to encourage the Association to render assistance on another occasion. If it be considered desirable by Guardians to take advantage of the services of a district nurse, the fee given to her employers should, at all events, cover out-of-pocket expenses.

### A WISE DECISION AT TRURO.

At the annual meeting of Truro District Nursing Association, mention was made of the fact that the Committee had been unable to take advantage of the offer of a home for the nursing staff on the condition that a third nurse, to take paying cases only, should live with the district nurses. The reasons given were the difficulty of obtaining the additional large sums of money necessary, and also that of combining nursing for charity with nursing for which payment is made. In our opinion, the second is quite a sufficient reason for declining a proposal which, however generously meant, is not in accordance with the principles on which the local branches of the Jubilee Institute are worked. We are sure that the people of Truro will give the little additional support which is essential to the employment of two Queen's nurses in the city; and it is obviously open to private enterprise to provide for the requirements of patients who are able to pay.

### PAYING PATIENTS AT BURY.

It was recently announced at a meeting of the Bury branch of Queen Victoria's Jubilee Institute that Mr. Henry Whitehead, ex-High Sheriff of Lancashire had presented the Association with a lease for 999 years of a large house in Manchester Road for a nurses' home and offices, the rent being fully secured. Mr. Whitehead has put the house in good repair, and on the strength of this most handsome present, it was decided to engage a fourth Queen's nurse. A nurse is also to be engaged for paying patients. We warn the Bury committee that the combination of district and private nursing under the auspices of one organisation is almost invariably attended by difficulties. They might profit by the example of Truro.

### SETTING THE LOCAL GOVERNMENT BOARD AT DEFIANCE.

In spite of the fact that the Local Government Board have expressed their regret that the Kidderminster Guardians have decided not to increase the nursing staff at the workhouse, and warned the Guardians that if any untoward accident should arise as a result of this decision they must be held responsible, no addition is to be made to the number of nurses. At the last meeting of the Guardians, the Chairman, having read the letter from the Local Government Board, which concluded with a significant reminder that the employment of pauper inmates of workhouses as nurses is prohibited by the Nursing Acts of 1897, alleged that the nursing staff is ample, and the next business was at once proceeded with. This is setting the Local Government Board at defiance, and bearing in mind the circumstances under which an inmate of the workhouse recently died, we think it is time that the people of the town made a protest. The Guardians do not meet again until January 10th, and by that date it should be made clear to them that the ratepayers of Kidderminster, however anxious they may be to escape unnecessary expenditure, do not wish to have their destitute sick poor exposed to the risk of dying from want of proper attention.

### MISCONDUCT AT A COUNTY ASYLUM.

At an inquest before the Berks County coroner on the body of a young woman who died in the County Asylum, Moulsford, evidence of a character very damaging to some of the female nurses was given. It was a question at the outset whether one of the nurses would not be found to have been guilty of an act of violence which caused the patient's death; but in the end the verdict of the jury was that the deceased died from hæmorrhage of the brain caused by excitement while being removed to the lavatory, and not from the assault committed by the nurse. As to the assault, however, the facts do not admit of doubt. The nurse herself stated at the inquest that she took the patient by the hair and shook her, with the result that the patient's head bumped on the floor, and that she asked two other nurses who were present at the time to deny that she used any violence. These nurses did as she requested them, and afterwards acknowledged to the medical superintendent that they had deceived him. Their obvious duty was, of course, to obey the rules and report the assault. We note that the coroner said that inquests in the asylum at Moulsford are seldom necessary, but that one nurse should pull the hair of a patient, and two others conspire with her to suppress the fact, indicates that there is room for improvement in the class of female attendants engaged in the wards.

### A WARNING TO NURSING HOMES.

An unsuccessful claim was made in Brighton County Court the other day by Miss Mary Canning, of a nursing home at Shoreham, for £50. The ground on which the judge gave judgment for the defendant was that no authority was given to pledge his credit. It was not suggested that the patient had not been properly nursed and treated in the nursing home. We are sorry for Miss Canning, if this means that she will lose money to which she is entitled from somebody, but we cannot, of course, go behind the facts which satisfied the judge, that the defendant could not legally be called upon to pay. In cases where an arrangement is concluded by a third person, and any question of liability is likely to arise subsequently, a written guarantee from the individual supposed to be responsible should be required before the admission of the patient, at any rate, if the patient is not recommended by a medical man.

### THE APPOINTMENT OF POOR LAW PROBATIONERS.

A curious question arose when the appointment of a probationer by the Wolverhampton Guardians was under discussion at their last meeting. Among the candidates who attended was one from South Shields, and a guardian asked her if she expected that she would stand any chance among local candidates. The chairman objected to the interrogation, and it was not answered; but after the candidates had left the room the guardian justified the question, because he contended that outside candidates were invited to come before the board, although they had no prospect of success. His view, however, was not in this instance warranted, for the South Shields candidate received the highest number of votes, and the local candidate the lowest. But issues of this character might be avoided if the Wolverhampton Guardians, instead of selecting probationers themselves, entrusted the duty to the superintendent nurse.

### CENTRAL MIDWIVES BOARD.

A meeting of the Central Midwives Board was held on Thursday last week. A letter from the clerk to the Privy Council with reference to the scheme of examinations as submitted by the board for approval was read, the suggestions made therein adopted, and the scheme referred back to the Council with their alterations and a change in the date of the first examination. This it is now proposed shall be in June and the number annually three instead of four, to meet difficulties which further consideration showed would result from the previous arrangement. The Privy Council's amendments included a provision that all examiners shall be men or women who are duly qualified medical practitioners, with power to appoint for certain portions of the examinations duly qualified women who are not medical practitioners, whose remuneration should be provided out of the funds payable to the examiners—with the consent of the Board. Also that the remuneration of an examiner should be not less than £2 2s. for each examination. In reply to a letter asking for some statement on the part of the Board as to the conditions upon which training schools for midwives would be recognised, the Secretary was directed to say that the Board could not at present formulate such conditions. A

number of letters were dealt with referring to the conduct of certain certified midwives, and a quantity of other correspondence was considered. The applications of 938 women for certification were approved, and their names were ordered for entry on the roll. This brings the total number enrolled to 11,476. The applications of Louise Margaret Hospital, Aldershot, and King's Norton Union Infirmary to be recognised as institutions for the training of midwives were passed, and the certificates of theDundee and Aberdeen Maternity Hospitals under section 2 of the Act were approved.

### COUNTY COUNCILLORS AND THE MIDWIVES ACT.

In Devonshire the County Council are delegating their powers under the Midwives Act to such Urban and Rural Councils which are disposed to accept them. Some of the ideas as to the requirements of the Act are curious. At St. Thomas District Council when the clerk notified that the County Council had vested the authority with the powers alluded to, one after another the alarmed councillors begged to be excused from acting on the committee for the supervision of midwives and the exercise of the powers conferred by the Act, but in the end a sufficient number of members agreed to serve.

### A GREAT MANCHESTER INSTITUTION.

At the annual meeting of the Manchester and Salford Sick Poor and Private Nursing Institution, presided over by the Lord Mayor, the account given of the work done in the twelve months was very striking. The district nursing staff now number 61, and during the year they attended 10,059 patients in their own homes and paid 245,344 visits. Unfortunately, though the Salford Home is self-supporting, there is a deficiency on the general fund, and a contribution of £130 towards the support of the district nurses' work was made from the private nursing department. The continued growth of the organisation attests its value, but the fact that money earned by the private nurses has to be voted in order to keep a charity in funds is a matter for regret.

### SOCIAL GATHERINGS FOR NURSES.

The first of the proposed little monthly social gatherings for nurses was held the other day at 34 Leinster Square, Bayswater. From 2.30 to 7.30 nurses were free to come and go as they were able, and during those hours nine availed themselves of the invitation. Garments provided and ready cut out gave occupation to some who liked work, and there was tea from 4 to 5.30 During the last hour and a half Miss Beyer, a Church of England Zenana Missionary from China, gave an informal address, and pleaded on behalf of the Mission Hospitals, which are in want of fully-qualified nurses to train the native girls. Any further information on the subject can be obtained from Miss Whitehead, at 34 Leinster Square, W.

### A NEW MODE OF RAISING PASSAGE-MONEY.

At the annual meeting of the Hong Kong Nursing Institution it was stated that during the year the two nurses terminated their agreement, their places being taken by two nurses from England. As the institution has not the money to pay the cost of passage necessitated by these changes, the committee got up a subscription-ball, the profits from which not merely covered the amount required, but yielded a balance to the good. This, however, was partly due to the fact that one of the late nurses accepted an appointment in the East, and, in her case, only half-passage had to be paid. The engagements of each nurse averaged 276 days in the year, and the working account balance remains on the right side. It was decided not to proceed with a scheme which had been prepared for the erection of a special building for nurses.

### THE QUEEN OF ROUMANIA AS NURSE.

Carmen Sylva, the Queen of Roumania, has been relating her experiences during the Russo-Turkish war in a North American magazine. The story of the sufferings of the wounded bears a strange resemblance to some of the accounts in the papers of the terrible distress undergone by the Russians in the present struggle. The little station of Cotroceni, where "the Princess"—as Carmen Sylva was styled by her patients—began her training had been turned into a refuge for the wounded on their way to other hospitals. Here the poor creatures came, groaning, even after being put to bed, "Oh, those carts! those carts!" where often they had been obliged to remain with gangrenous wounds, frostbitten feet, without food or drink, jolting over the roads for five days. The Princess usually rose at four, after only a few hours' rest, for so much had to be done, but she says that she was nobly aided in her nursing efforts by the women of Roumania. Day and night they were to be found at their posts, giving all they had, whether they could spare it or not, and helping to the utmost, not because they were trained nurses, but because there was no Red Cross Society then to organise. Young girls were allowed to write letters for the soldiers, to distribute the food, and to make cigarettes; whilst the Princess and other ladies—many of whom had learnt first aid of necessity on their own estates in the country far removed from a doctor—assisted the doctors in the operations and tended the patients.

### THE JUNIUS S. MORGAN BENEVOLENT FUND.

The secretary of the Junius S. Morgan Benevolent Fund desires to acknowledge, through our columns, the receipt of £1 1s. from an anonymous donor who signs herself "A Sister." This kind contributor has sent a guinea to the fund for several years.

### SHORT ITEMS.

A number of valuable gifts have been presented to Miss McCord on the occasion of her departure from Queen Charlotte's Hospital. They include a handsome brooch from the sisters, a bracelet, and a dressing case, the latter being presented by the committee of the hospital.—The Mayor and Mayoress of Portsmouth visited the wards of the workhouse infirmary on Christmas morning and greeted the patients and staff most heartily. The medical superintendent, on behalf of the medical and nursing staff, presented the Mayoress with a framed picture "The Apotheosis of Sairy Gamp," as a memento of the day and as a small recognition of the great interest shown by her in the training school.

### The Nursing Outlook.

"From magnanimity, all fear above;
From nobler recompense, above applause,
Which owes to man's short outlook all its charm."

### THE SCHOOL NURSE.

About eighteen months ago we expressed a hope that, when the then School Board of London had disappeared, one of its good works, that of the maintenance of the visiting nurse for elementary schools, would remain and prosper. The school nurse has been a triumphant success, for she has by her excellent work introduced personal cleanliness amongst children to a degree which at one time appeared to be impossible. The Medical Officer of the late School Board in his last report insists that much of the disease, and temporary or even permanent injury to health, suffered by children is the result of personal uncleanliness. A very considerable part of the defective vision noticed amongst the scholars is due to this, resulting in laceration of the cornea, inflammation of the eyelids, and in chronic conditions which are aggravated by neglect; with wounds or sores, chilblains, discharging ears, and so on, whilst dirt and neglect aggravate the conditions. Personal cleanliness in the children is one of the first of sanitary considerations, and the most important object to be attained in consequence.

The work of the school nurse has extended and increased in interest since the office was originally created. At first only three nurses were appointed in London to visit schools, and draw attention to ringworm and other conditions; the work varied from day to day. Sometimes every child in the class was examined, sometimes only those selected by the teacher, and generally cases pointed out by the nurse after her formal inspection of the class. Under the later developments, a nurse is attached to a school; she examines frequently and repeatedly all scholars, and she visits and remonstrates with parents who may remain obdurate. As her work progressed, and conditions of tolerable cleanliness were made to prevail, it was found that the improvement could be made permanent by regular supervision on the part of the nurse and the teachers. In October 1903 the number of nurses employed to visit the schools was doubled; in July last it was again doubled, raising the total number of nurses engaged to twelve; and the Education Committee of the London County Council will, it is believed, appoint a permanent and sufficient staff of nurses for the whole of the London schools.

This important improvement is mainly due to the excellent work done by the London School Nurses' Society, the final report of which has just been issued. It would be difficult to exaggerate the benefits which are likely to result from it to the health and comfort of the children of the humbler classes throughout London. We are glad to note that the Society appears to hold that 12 nurses in the ordinary day-schools are sufficient for the work at present. No doubt they will be increased in due course, should such a step be found necessary. At the outset the school authorities were not alive to the importance of appointing school nurses, and in consequence, as is so often the case in England, private charity has shown the way, and the public authorities have, as a result, now undertaken this duty themselves, and will no doubt provide that the staff maintained shall be adequate to fulfil to the utmost the purposes for which the school nurse was originally instituted.

We think that nurses as a body ought to be proud that a few of their sisters should have been able in so short a time to have accomplished so much for the children of London. Each year new opportunities are given to nurses, and fresh fields of labour are opening to them, so that apart altogether from the attraction of the work itself, a nurse of intelligence has increased interest offered to her in her profession by the variety which progress in science and hygiene provides, by opening up new opportunities to her for useful service.

No doubt the ever-extending welcome which competent nurses meet with must be placed largely to the credit of the work done by the best nurses everywhere in these days. The first school nurses, by their devotion, tact, and zeal, have not only conferred great benefit upon the community, but they deserve special credit for having so conducted the difficult task assigned to them that they have secured the cordial recognition of public authorities and the gratitude of the parents with whom they have been brought in contact. It is gratifying, too, to recognise that the work itself must have a permanent effect for good, not only upon the health, but upon the habits and character of children and parents alike. Who shall estimate the amount of happiness which the work of the school nurse brings to childhood, or of the gratitude which the removal of the discomforts incidental to uncleanliness must everywhere excite? All honour, then, to the school nurse. May she continue to abide and flourish for ever.

It would not be just to conclude this article without expressing the indebtedness of the public to Lady Windsor, the president, to the vice presidents and committee of the London School Nurses' Society, without whose efforts the school nurse in London, at any rate, would probably not yet have been tried, much less established upon so satisfactory a basis as she stands to-day. Great praise is due to the honorary secretary of the society, Miss Susan Lawrence, who is also a member of the sub-committee of the Medical Officer's Department of the late School Board for London. Miss Lawrence's public work is well known and widely recognised. She has done much for education, as well as for the children themselves, and to her in large measure we owe the successful establishment of the school nurse. All who have had anything to do with this work have the fullest reason to be satisfied with the results of their exertions.

### Lectures upon the Nursing of Infectious Diseases.

By F. J. Woollacott, M.A., M.D., B.S.Oxon, D.P.H., Senior Assistant Medical Officer, Park Hospital, Metropolitan Asylums Board, Hither Green.

### LECTURE XI.—DIPHTHERITIC CROUP.

(*Continued from page* 168.)

*Tracheotomy.*—The same general preparations should be made as for any other operation; but some points need special attention. The tracheotomy tubes should be all ready threaded with the tapes, so that no time may be lost, and the supply of cotton wool swabs must be plentiful. A firm pillow is necessary for placing behind the patient's neck in order to throw the windpipe prominently forwards. It should consist of a tightly rolled up draw sheet or a small sandbag. Brandy and a hypodermic syringe should always be at hand. An anæsthetic may or may not be given. As it is often undertaken hurriedly, the nurse may be called upon to help during the operation itself. If directed to hold the patient and restrain his struggles, she should take firm hold of his arms, one in each hand, and hold down the legs by leaning on them. The arms should be held to the sides of the trunk and the body kept as straight as possible. No pressure must be made on the chest or abdomen. If holding the head she should fix it firmly between her hands, keep it quite still and in a straight line with the body, at the same time stretching the neck over the pillow. When the trachea is opened a quantity of mucus or membrane is usually expelled, and there may be considerable bleeding from the wound. All this should be swabbed away as rapidly as possible, and we should never allow it to be drawn back into the windpipe. It is to be removed by firm sweeping movements of the swab at the end of each cough or expiration. The best dressing is composed of two layers, fixed together by a few stitches. The inner one, next the skin, should consist of boracic lint; and the outer of jaconet, which being waterproof prevents the discharges from the tube from soaking through and fouling the wound.

After the operation the patient should be put into a warm bed, where he will often go to sleep at once. In the first few hours he should be disturbed as little as possible. The subsequent nursing is of the greatest importance, and the case must be carefully watched and never left. The temperature of the room should be 70° F. or a little lower. A steam-tent or half-tent is rarely necessary, but is occasionally of service. On a table by the bedside should be kept the following instruments: tracheal dilators, a spare tracheotomy tube of the same size as that in use, ready fitted with a pilot and tapes, forceps and scissors, as well as materials for cleaning the inner tube, swabs, and fresh dressings.

One of the chief duties of the nurse is to keep the inner tube from becoming blocked. With this object in view it should from time to time be removed gently, and quickly cleaned by means of strips of lint or feathers dipped in a warm carbonate of soda lotion, and then at once re-inserted. No rule can be laid down as to how often this should be done, as it depends entirely on the amount and nature of the secretions from the trachea. Both in cleaning the tube and in removing or replacing it the greatest gentleness must be observed, otherwise it will be bent or otherwise damaged. A skilful nurse will often manage the whole business without waking the patient; but the use of force, however slight, will cause pain and discomfort. If it fit well the inner tube will fall in with its own weight, and the catch should then be carefully adjusted with forceps. On no account should feathers be poked down the tube while still in the patient's neck. Such a practice is dangerous, dirty, and useless.

The food should be light and given every few hours at first. If it causes cough or if liquids return by the wound, nasal feeding must at once be begun.

It occasionally happens that after tracheotomy little or no improvement takes place. This usually means that the membrane has extended into the lower part of the trachea or even further along the air passages. Treatment then becomes very difficult. Coughing should be encouraged in order to loosen and expel the membrane, and for this purpose an alkaline spray or the instillation of creasoted oil may be of service. The tube is nearly always dry and sticky owing to the scantiness of the secretion, and the use of the steam-kettle may afford considerable relief. Very frequently, however, in these cases treatment is unavailing and the patient succumbs either from pneumonia or exhaustion. At times, but fortunately very rarely, sudden and urgent symptoms of suffocation set in. Such occur if the tube become blocked or slip out of the trachea; and the patient's life may then depend on the promptitude and skill of the nurse. She should remove the inner tube, and, if no relief follow, cut the tape and remove the outer one as well, sending at the same time an urgent message to the first doctor that can be found. If the breathing be relieved nothing more need be done, but if not she should take the dilators, insert them into the trachea and hold it open till help arrives. Under no other circumstances should the tapes be interfered with, or the outer tube removed without express instructions.

Under favourable conditions the outer tube is best left undisturbed for about two days after the operation. It is then changed by the doctor or, if the patient seems comfortable without it, an attempt may be made to dispense with it altogether, though this is rarely possible at so early a stage. After its removal the patient should be soothed in every possible way, for if he gets frightened or fretful his breathing will soon become embarrassed and necessitate its reinsertion. He should be talked to and encouraged to play with his toys, and it is a good plan to get him to blow about a feather, so that breathing by the mouth may be restored as soon as possible. The wound in the neck should be lightly covered by a piece of gauze, loosely fixed in position by tapes. It will gradually heal and the voice will return. Both when the tube is in and afterwards the nurse should be on the alert to swab away any membrane or mucus that may be coughed up. In exceptional cases the tube has to be worn for a considerable time, but with perseverance its ultimate removal can nearly always be effected.

*Intubation.*—This operation may be of use in comparatively mild cases, and before symptoms of exhaustion have appeared. No anæsthetic is required. The patient is wrapped round with a sheet to restrain his struggles, and his mouth held wide open by a gag. He is either sitting upright on the nurse's lap, or else lying on his back. To the intubation tube a thread is sometimes attached, the end of which, after the operation, is brought out of the mouth and fixed to the cheek by strips of strapping. In this case splints must be fixed to the child's arms, so that he may be unable to raise his hands to the face. As regards the after treatment, food must always be given by the nasal tube. Coughing should be encouraged, and for this purpose sips of water are useful. If signs of urgent distress arise, as they occasionally do, the tube is probably blocked and must be pulled out at once by means of the thread. The doctor should, of course, be informed immediately. If all go well the tube may be dispensed with in a few days and normal breathing restored. Not infrequently, however, the operation fails to afford relief immediate or permanent, and sooner or later tracheotomy is necessary. For this and other reasons intubation is regarded by many medical men as an unsatisfactory and dangerous operation, and it is certainly far less generally useful than tracheotomy.

### Christmas in the Hospitals.

BY OUR OWN COMMISSIONERS.

In our Christmas Number we were able to give a striking demonstration of the manner in which the spirit that has for some years prevailed in our great voluntary charitable institutions has permeated the fever and other hospitals. It must always be remembered that the practice of making Christmas a happy period for victims of disease and accident originated in establishments supported by the voluntary offerings of the public, and the fact that there has been in various directions a remarkable development of the movement renders it all the more desirable that there should be no slackening of effort in the pioneer hospitals. There is always a danger lest, as time goes on, these observances of the most admirable habit should degenerate into a perfunctory formality, and though this is diminished in hospitals owing to the frequent changes among members of the staff, with the greater chance of exciting new enthusiasm, it is one to be guarded against. Happily, as the reports of our Commissioners who have visited the various hospitals this year during the progress of the entertainments abundantly show, custom has not so far either staled the infinite variety of the resources, or dried up the fount of sympathy, upon which the sick and suffering are now accustomed to depend in order to reconcile them to absence from home at Christmas. It may be doubtful whether in the vast majority of cases the patients would have half so many comforts at home as in hospital, but it is natural that they should desire to be among their own people at such a time, and the carefully organised, and excellently carried out, plans for their gratification, when all the world is keeping high festival, must be an immense alleviation even of a hard lot. As long as this alleviation continues to be afforded in the ungrudging and generous spirit which has been no less clearly manifested during Christmas 1904 than on previous occasions, the supporters of our hospitals will have the satisfaction of feeling that, if they find the money to keep them going, the physicians, the surgeons, and the students, the matrons, the sisters, and the nurses, gladly supplement, when they have the opportunity, their professional duties by the kindly acts of service which are the hall-mark of genuine interest.

### Charing Cross Hospital.

Christmas Day was ushered in at Charing Cross Hospital by carols, sung on Saturday evening by the nurses in the wards. On Sunday morning carols by the choirs of St. Martin-in-the-Fields and the Mission Church attached took the place of the usual sermon. This was followed by the Christmas dinner. Monday was the day devoted to the ward teas and concerts, when each bed had its group of visitors gathered round it, and some excellent and amusing musical programmes were given. The clever imitations and songs by Mr. Sampson, eulogistic of "Pinkerton's Purple Pills," were especially appreciated. Every ward was elaborately decorated with pink-and-white chrysanthemums, smilax, ribbons, plaited paper or flags, and the many dainty fairy lights which sparkled among the greenery were lent by Messrs. Clarke, while other firms were equally generous in contributions to the wards. The Christmas dinner for sisters and nurses took place on Monday, and on Tuesday there was a tree for the children in the Levy Ward. Presents for all the patients were provided by the hospital, and distributed on Christmas Day.

### Guy's Hospital.

A cleverly illustrated programme was handed to visitors to Guy's Hospital on Monday afternoon, by means of which the troupes of students could be followed on their tour through the various wards. The most heterogeneous costumes had been called into requisition this year, and at unexpected corners one was liable to encounter a North-American Indian, a Golliwog, a South Sea Islander, or an astonishingly muscular hospital nurse playing bones. Every ward was daintily decked out with graceful screens of evergreen, and in Cornelius Ward a leaf had been taken out of a Regent Street shop, the screen formed of narrow coloured ribbons in the shape of a spider's web, with a large luminous spider lurking in its meshes. Useful presents had already been distributed to all the patients, either on Christmas Day or on Monday. At two o'clock tea was provided for about 500 poor children of the Borough, and the help of several of the Metropolitan police had to be called in to keep the gates—on which many little uninvited guests hung enviously—as well as to maintain order within. Amid the sound of tin whistles, shouts of "Bill Bailey," and the strong odour of oranges, presents were distributed, followed by a cinematograph. After a varied entertainment, the little guests departed, each carrying a parcel of biscuits, tea, a loaf of bread, an orange and a bun. Ward concerts were continued throughout the week, and Thursday was the day chosen for the Christmas tree and cinematograph entertainment in Martha Ward, to which all the children in the hospital were invited. Many of the gifts were supplied by Mrs. McMillan.

### London Hospital.

In order to avoid interference with the holidays of the residents and students the entire Christmas programme at the London Hospital was carried out on Saturday. The day began with the singing of carols by the sisters and nurses, who visited each ward. Then came presents, distributed by Father Christmas and clowns, followed by dinner. During the afternoon a well-organised series of entertainments was gone through by the students and their friends, making, in all, fourteen troupes. The students' bands included the Pirates, realistically costumed and armed with large pewter drinking vessels; the Kilties, a good presentment of Highlanders in somewhat motley tartan, who played mock instruments with great effect, and gave a stirring performance of "Bill Bailey"; the Aliens, the Knockabouts, Box and Cox, and the Freaks; while among the visitors were the Oxford House Minstrels, Marah Raj and Maf, Punch and Judy, Mr. Julian Ross, Messrs. Debenham and Ward, Mr. J. H. Woods, Mr. Arthur Hilton, and Mr. Relph. By means of clearing some of the wards and filling up others with all those patients who were sufficiently convalescent to enjoy the performances, a great variety of entertainment was possible, and many of the troupes gave as many as eight performances between 3.30 and 7.30, when they visited the out-patients and repeated their amusing antics once more. All the wards were prettily decorated, and festive teas began at 3.30 and continued at intervals during the afternoon and evening. There was a Christmas tree in Queen Ward, the fruits of which were left on the branches until Monday, so that the Sunday visitors to the little patients might see the ward at its best.

### London Temperance Hospital.

Christmas at the London Temperance Hospital, in the Hampstead Road, was the happiest day in the lives of many of the poor folk who live in the immediate squalid neighbourhood, and who happened at this time to be lying ill in the wards. Splendid dinners of turkeys, plum puddings

### CHRISTMAS IN THE HOSPITALS—*Continued.*

and jellies were provided for those who could enjoy them; the afternoon was devoted to a tea to which each patient invited a friend; the men smoked cigars presented by the staff; and the sisters, nurses and doctors sang and played and entertained them right royally. At six o'clock an entertainment, got up by the matron, was given, and all who could with safety be moved were carried on stretchers or in chairs, and "encores" were the order of the evening. On New Year's Eve a huge Christmas tree, brilliantly illuminated with electric light, and loaded with presents for everyone, will take place, followed by an entertainment consisting of "tableaux vivants," got up by the matron.

### Middlesex Hospital.

Christmas was celebrated on Saturday at the Middlesex Hospital when "presents for everybody," chosen by the lady superintendent, were hung on a large tree in the board room, with an array of "Truth dolls" standing or sitting at its roots. The wards were tastefully decorated, one of the most original features being "Prehistoric Japan," in which, on a snowy landscape, not only a sea-serpent and an ichthyosaurus, and other dangerous-looking animals, but an aboriginal man (or possibly an ape) appeared. This was in May ward. The two Christmas trees in May and Percy wards were stripped of their many fascinating fruits on Tuesday and Wednesday respectively. One little patient is spending her third Christmas in May ward, and evidently enjoying it as much as its predecessors; and this ward also boasts a "show baby," who endures almost more than her share of baby-worship with great resignation, and opens her eyes widely at the wonderful decorations, flowers, and fairy-lamps, by means of which the wards have been transformed into fairy-land for the time being.

### Queen Charlotte's Hospital.

The plan of giving each sister, nurse, and member of the domestic staff at Queen Charlotte's Hospital the value of a theatre ticket was adhered to this year, thus ensuring a choice of entertainment and enabling the recipients to arrange pleasant meetings with friends. About 90 of the nurses and pupils gathered at the Christmas dinner on Monday, although, owing to stress of work in the district, several nurses were unable to be present. The wards were tastefully decorated, the mass of evergreen, however, being reserved for the chapel, where there were some beautiful white chrysanthemums on the altar and font. A pretty effect was produced by threading berried holly in the ironwork of the screen surrounding the lift, upon which a motto in scarlet and white had been suspended. A concert for the sisters and nurses has been arranged for Tuesday next, at which, in order that the wards may not be left unattended, the staff will be present in detachments.

### Royal Free Hospital.

The pretty custom of carol-singing by the students was adhered to this year, when the wards of the Royal Free Hospital were visited in turn on the Monday and Tuesday preceding Christmas Day. On Boxing Day each patient received two or three presents, and at tea time family parties gathered round the beds, and musical entertainments were provided by the sisters and nurses and their friends. The wards were prettily decorated with flowers, evergreens, and fairy lamps. One side of the hospital buildings being closed for reconstruction, a party was given by Sister Boys in the lecture-room, where about 20 children who have been patients were entertained to tea, music, and a Christmas-tree. For this a number of toys, stockings filled with sweets, etc., were provided by *Truth* and the *Daily Mail.*

### St. Bartholomew's Hospital.

Christmas was celebrated at St. Bartholomew's Hospital on Monday afternoon, when turkeys and plum puddings were provided, followed by sweets and fruit. Great pleasure was created in Stanley Ward by the fact that cups and saucers were used at tea-time in place of the ordinary mug, and that the patients were allowed to sugar their own tea. There was a Christmas tree in this ward, too, and every patient received, in addition to gifts from its branches, several useful garments. All the patients were decked with red bows. A conjuring entertainment was provided after tea, and after plantation songs from some of the staff, the ward settled in for the night at 6 30. Another ward which boasted a magnificent electric-lighted tree was Lucas, and fairy lights, evergreens, and coloured paper had been used with very gay effect. A centre of interest in this ward was a small table where half-a-dozen tiny patients were having tea. While there are no organised entertainments at this hospital, a little quiet amusement is always provided by the sister in charge of each ward, and has the approval of the medical and surgical staff, and the singing of carols on Christmas Day was greatly appreciated by the patients.

### St. Thomas's Hospital.

Although Christmas teas took place in the wards of St. Thomas's Hospital on Saturday, there were no organised entertainments until Tuesday, when the "Hospital Pierrots" began a tour of the wards, to be continued up to Saturday. The performers included Herr Follicle, Bill Harzia La Bella Donna Cascara Sagrada, the Blood Count, General Peritonitis, Mistur Alba, Pa Pilloma, Sir Vical Vane, Count Terry-Forceps, Major Operation, and Phlebitis, the last being a small fox terrier, wearing a muslin frill and led by a string. Some very good part songs and solos were given, and the duet "Where are you going to, my pretty maid?" by Herr Follicle and Sir Vical Vane, was greatly enjoyed. In Clayton Ward a little patient sang a topical song, "Mother, come and fetch me home," learnt at the Convalescent Home at Broadstairs, in which the lines

"Here comes nurse with a red-hot poultice,
Claps it on and takes no notice,"

caused much amusement. The wards were hung with Chinese lanterns, presents were given throughout the hospital, and smoking was allowed in the men's wards. There were Christmas trees in the children's and female surgical wards and in the casualty department.

### St. John's Hospital.

A very successful entertainment was given to the in-patients of St. John's Hospital for Diseases of the Skin, Uxbridge Road, on Saturday evening. The first part of the programme consisted of a one-act comedietta, by Walter Gordon, entitled "Dearest Mamma," in which three of the characters were acted by nurses. The second part included pianoforte solos, songs by Nurses Weir and Viggars, the Misses Dorothy Law and Gladys Churchouse, Mr. Charles Morton, and others, recitations, musical sketches, sleight of hand, and a ventriloquial interlude, introducing "Marmaduke." Only two patients were unable to leave their beds, and all were present at the performance, which lasted from 6 p.m. to about 9 p.m., concluding with a dance for the nurses. Each patient received a present, and was allowed to have a visitor to tea. On Christmas Day the children—of whom there are about a dozen in the hospital—had a tree. The wards were tastefully decorated with evergreens, flowers, and Chinese lanterns, and every one lent a hand to make the festival a happy one.

SAMARITAN FREE HOSPITAL.

Christmas was celebrated on Sunday at the Samaritan Free Hospital by the singing of carols and festive services conducted by the chaplain. On Monday the patients, nurses, and domestic staff had their Christmas dinner, the patients afterwards entertaining their friends to tea. This was followed by an impromptu concert, and carols were sung to the accompaniment of a piano, which was moved into the central hall. Chinese lanterns, berried holly, and choice flowers had been used in the decoration of the wards. On Tuesday tea was provided for visitors at four o'clock, after which a musical entertainment was given in the Board Room, at which a number of the patients and most of the staff were able to be present. A song by Mr. da Costa Ricci opened the concert, and this was followed by "The Knights," by Miss Wakefield, violin solos by Mr. E. Haarbleicher, piano solos by Miss Cochran, recitations by the Rev. H. Russell Wakefield (Mayor of Marylebone and chaplain of the hospital), and other items. Presents were distributed at the close of the evening to patients, sisters and nurses, and the domestic staff.

WESTMINSTER HOSPITAL.

Nothing occurred at Westminster Hospital to mar the Christmas celebrations on Saturday, except that the dainty little fairy, dressed by the incurable patients in muslin and silver gauze, was frightened by the "animal" who accompanied Father Christmas and the sailor troupe, with a piano, on their rounds, and refused at first to give out the presents; she soon recovered, however, and enjoyed the fun as much as anyone. The bran-tubs, as usual, called forth all the inventive powers of the sisters, and Humpty Dumpty, sitting on a wall, his oval body filled with gifts, which had to be fished for through his brain, was perhaps the most original form they took this year. A very pretty "tub" was in the shape of a chrysanthemum, standing about three feet high on a sturdy stalk. This was in Arden ward, where the decorations were particularly graceful, and included many choice blossoms. Tall palms, elaborate lamp-shades, and dainty paper baskets on the small tea-tables helped in the general effect, which was heightened in all the wards by innumerable fairy-lights. An excellent programme of songs and music, with a sketch called "Our Toys" (the scene of which was laid in the nursery), was given in the evening. The sisters and nurses had their Christmas dinner on Monday, and their At Home, when each invited a guest, on Tuesday, from 7.30 to 10.30.

(*To be continued.*)

### Nursing in the Russian-Dutch Field Hospital at Taolatsckao.

THE following letter is a translation from the letter of a nurse published in the *Petersburger Zeitung*, which was written in October.

"On the 7th there were handed over to us, quite unexpectedly from an ambulance train, thirty severely wounded men who could not be sent any further. In that train were 1,300 wounded, travelling without any comforts, they were accompanied by a doctor and two evangelical sisters, and were being taken from the battlefield to Karbin. Our poor wounded were in a terrible condition, many had had nothing to eat for some days; they lay, stretcher by stretcher, the whole length of the ward, and waited with touching patience until the sisters fed them one after the other, then washed them, changed their clothes, and got them ready for dressing. It was late at night before they were at last in their beds, their wounds dressed, clean and satisfied.

PATHETIC INCIDENTS.

"Almost all were suffering from hopelessly dangerous wounds in the abdominal and thoracic regions. One had received a shot through the mouth, as he opened it to cry hurrah, and it had gone out again through the back of his head. The unfortunate man had lost 13 teeth in the process. In another case, the whole of the lower jaw had been destroyed. In yet another the ball had passed through the cheek and out at the neck. Six men died during the next few days, and there are still several who are approaching their end, and for whom human skill can do nothing more. One of these an attractive lad of 24 years, with a fresh coloured face, received a rifle shot in his side, the ball remaining in the bladder. The poor boy suffers terribly, for gangrene has appeared in one foot, and in spite of every effort it is getting worse. One can hardly believe that this pleasant, kindly youth is doomed to death. Yesterday I had to write a letter home for him; when I read the letter to him and his neighbour they both began to cry bitterly. Both are married and have children, and both must die.

A TERRIBLE CASE.

"The most terrible wound of all was that of a poor soldier who was released yesterday from his sufferings. A shell had torn a great hole in his right leg by the hip, shattering the entire bone; a large piece of flesh had also been torn from the other leg. Under the knee there was a perforating wound. And all these wounds were suppurating to such an extent that gangrene had begun in several places. What the poor man must have suffered! His dressing always lasted more than an hour. During the last hours before his death he cried persistently for 'Sister,' hung on to my hand, or threw his arms round my neck. To-day I wrote to his old mother about his death, and told her that I would bring the watch her son had left behind myself or send it if an opportunity occurred.

FUNERALS DAILY.

"Since Sunday we have had funerals every day; in three days there have been eight deaths. High up on the hill top many crosses stand side by side. Yesterday we received from a train three typhoid patients; one died after a few hours, a second on the following day, and the third is not likely to recover. Seventy beds are invariably occupied. There is a great deal of work, and one sister or the other is always ill; even I knocked up the other day for the first time—it must have been the result of over-fatigue. The male members of our corps are also very dilapidated. I was so weak that I could hardly hold myself up. On the third day, when I had just begun to recover, there arrived a large transport of wounded, and I had to forget everything else and set to work. In the evening I collapsed again.

NIGHT DUTY.

"Now I am really better. To-day, with the doctor's permission, I have been able to take night duty for the first time. It is not a light undertaking just at present. A typhoid patient, whose condition has suddenly become worse, is a great anxiety to me. On all sides one hears groans, moaning, and coughing. The whole night is spent in wandering from patient to patient in the great ward. I have a man to help me, and we two have quite enough to do. Yesterday we received a telegram asking us to prepare for an even greater number of patients."

### Appointments.

[No charge is made for announcements under this head, and we are always glad to receive, and publish, appointments. The information, to insure accuracy, should be sent from the nurses themselves, and we cannot undertake to correct official announcements which may happen to be inaccurate. It is essential that in all cases the school of training should be given.]

ADDENBROOKE'S HOSPITAL, CAMBRIDGE.—Miss Susan M. Adams has been appointed matron. She was trained at the David Lewis Northern Hospital, Liverpool, where she has since been sister of a medical ward, housekeeping sister, and assistant matron.

BARNSLEY UNION INFIRMARY.—Miss M. A. Foster has been appointed nurse. She was trained at St. George's Infirmary, Fulham Road, London. She has since been head nurse at Plumstead Infirmary, sister at St. Pancras Infirmary (South), and superintendent nurse at Rochford Infirmary, Essex. She holds the L.O.S. certificate.

BETHNAL GREEN INFIRMARY, LONDON.—Miss Rosa C. B. Watts has been appointed superintendent nurse. She was trained at the Poplar and Stepney Sick Asylum, where she has since been staff nurse, ward sister, and theatre sister. Subsequently she was night superintendent at the Blackburn Infirmary, and she has also done private and district nursing. She holds the L.O.S. certificate.

BIRMINGHAM CORPORATION.—Miss C. J. Bement has been appointed lady inspector of midwives for the city of Birmingham. She was trained at Lambeth Infirmary, where she was subsequently staff nurse and for two years assistant midwife. She has done private nursing in Kent, and has since been sister of the maternity wards and nurses' home at Kensington Infirmary. She holds the L.O.S. certificate.

CAMERON HOSPITAL, BOROUGH OF WEST HARTLEPOOL.—Miss Leavis has been appointed matron. She was trained at the Royal Hospital, Belfast, and at Portsmouth. She has since been matron of the Fever Hospital, Newtownards, senior nurse of the Hartlepool Nursing Guild, sister at the Royal Infirmary, Wigan, and matron of the Private Hospital, West Hartlepool.

EAST GREENWICH UNION INFIRMARY, VANBRUGH HILL, S.E.—Miss Lucy H. Beard has been appointed matron. She was trained at Crumpsall Infirmary, Manchester, where she has since held the posts of ward sister, night superintendent, and second assistant matron.

HAMPTON ISOLATION HOSPITAL.—Miss Mildred Pride has been appointed nurse-matron. She was trained at the City Hospital, Grafton Street, Liverpool, where she has since been staff nurse. She has also been nurse at the Sisters' Hospital, St. Albans, and private nurse at Whitby.

ISLINGTON WORKHOUSE.—Miss M. W. Taylor has been appointed night maternity nurse. She was trained at Stockport Union Infirmary, and has since been assistant nurse in the maternity wards at Lambeth Workhouse. She holds the L.O.S. certificate.

LUFTKUR OPEN-AIR SANATORIUM.—Miss Helena Atthill has been appointed matron. She was trained at Middlesex Hospital and the Rotunda Hospital, Dublin. She has since been night sister at Stafford General Infirmary, sister at Monsall Hospital, Manchester, lady superintendent at Dover Hospital, and lady superintendent at the Invalid Home, Davos Dorf. She holds the L O.S. certificate.

NELSON DISTRICT HOSPITAL, NEW ZEALAND.—Miss Edyth M. Davis has been appointed matron. She was trained at West Ham Hospital, and has since been sister at the Children's Hospital, Belfast, sister at the Children's Hospital, Sheffield, matron of Loughton Hospital, Essex, and matron of the Provincial Police Orphanage, Redhill. Subsequently, she has been doing private nursing in Auckland, and acting as sister at the Government Sanatorium, Cambridge, New Zealand.

### Our Christmas Distribution.

THE following letters have been received by us from the recipients of parcels of garments which we were able, through the kindness of our readers, to distribute.

The Secretary of the Royal Free Hospital, Gray's Inn Road, W.C., writes:—"I beg to acknowledge the receipt of a parcel of clothing for the poor patients of this Hospital, and would ask you to accept the best thanks of the Committee for the gift, which will prove most acceptable in our wards."

The Matron of the City of London Hospital for Diseases of the Chest, Victoria Park, writes:—"Very many thanks for the parcel of clothing which you have been good enough to send to the hospital, and which I assure you will be greatly appreciated."

The Matron of Northwood Hospital (branch of Mount Vernon Hospital for Consumption, Hampstead). Middlesex, writes:—"I am desired by the Committee of Management to thank you for the very kind present of clothing for the patients, which is much appreciated."

The Matron of Guy's Hospital writes:—"Thank you very much for the useful present of garments you have sent to the hospital. They will be much acceptable to some of our poor patients. Will you please thank your readers for their kindness in remembering us?"

The Matron of the West Ham and East London Hospital writes:—"This is the second year that you have been kind enough to send some useful garments for our patients. I am most grateful and know how thoroughly the gifts will be appreciated by our sick ones. They nearly all come from very poor homes, and look forward for their Christmas gift of some suitable garment."

The Matron of the London Temperance Hospital writes:—"I beg to thank you and the readers of THE HOSPITAL for your parcel of garments for our patients."

The Matron of the Metropolitan Hospital writes:—"Please accept our very hearty thanks for the useful parcel of clothing, etc., that you have so kindly sent to the patients in this hospital; they are all so good and well made, and just the things that are most suitable for the poor, and we appreciate your gift very much, and beg to thank you most heartily for your kind remembrance of us."

The Matron of North-Eastern Hospital for Children writes:—"Please accept our thanks, and to the 'readers' also, for the clothes and dolls you so kindly sent us for the patients. I am sure that they will be greatly appreciated."

The Matron of the New Hospital for Women, 144 Euston Road, N.W., writes:—"I beg to acknowledge, with many thanks, the parcel of clothes for the patients."

The Lady Superintendent of the East End Mothers' Home writes:—"Will you kindly convey to the Editor and readers of THE HOSPITAL our most grateful thanks for the very useful garments they have so kindly sent for the patients of the home? The majority of the women are so poor, and their clothing so pitiable, that we are thankful to be able to get them a warm garment, and to send the babies home warmly clad."

### Everybody's Opinion.

[Correspondence on all subjects is invited, but we cannot in any way be responsible for the opinions expressed by our correspondents. No communication can be entertained if the name and address of the correspondent are not given as a guarantee of good faith, but not necessarily for publication. All correspondents should write on one side of the paper only.]

### WOMEN ON HOSPITAL BOARDS.

"E. C." writes: There is no doubt in my mind that, taken *en masse*, men are intellectually superior to women. But that women are not capable of sitting on hospital boards is an opinion that, I think, few men who work with women will endorse. Obviously there are incapable women, perhaps more than there are incapable men. But Lady Hermione Blackwood has made her point in alluding to Florence Nightingale and other women reformers. Some women have administrative ability and organising powers. They have, as a rule, more unselfish zeal, and more insight than men. Naturally only women possessing the right qualities should be elected; I venture to think they would satisfactorily replace some of the masculine members one has known on boards. It goes without saying that the female *dilettante* should not be chosen any more than the male amateur. If a woman with a knowledge of hospital work can be found, so much the better. But, at all events, she will possess a knowledge of household work and management, and the perceptive faculties which make her quick to perceive whether "things are going smooth." Hospitals are not all on a large scale, as you appear to imply in your note to Lady Hermione's letter; matrons, though an admirable race, are human, and, moreover, would in some cases I know, welcome fellow-women on the governing body. Speaking as a hospital nurse, I can remember reflecting—after the dear old board gentlemen had gone one of their regular rounds, digging into the potatoes with a fork and asking the patients whether they were satisfied—that a woman would have seen through to the truth that lay behind the answers, and know, for instance, where to look for wastefulness. Look at the board book of a provincial hospital—"Found all comfortable and satisfactory; and so on." Is this a farce? If so, stop the custom. If not, surely women could perform this duty better. It is not a question of looking out for mismanagement, or attempting to thwart the matron. New ideas and fresh points of view would be a help to an open-minded woman. Matrons, though selected, as you say, for their undoubted capacity, sometimes get their heads down with the bit between the teeth. Dare an irate governor dash at the bridle? I do not know; but possibly Lady Hermione Blackwood is right in counting on their chivalry to women. To sum up, I venture to think that on a hospital board you require the knowledge of finance and business, the breadth of view and moderation, of men as well as the knowledge of detail, imaginative sympathy, and devotion to work of women."

### VOLUNTARY AND COMPULSORY REGISTRATION.

MISS E. GLOVER, honorary secretary, Victorian Trained Nurses' Association, Melbourne, writes:—

Referring to an article in THE HOSPITAL of September 17th, on "Quality of Training," voluntary registration, such as exists in Victoria for its 1,000 nurses could not apply to Great Britain with its 80,000. There are enormous obstacles to be overcome before a like condition could be established in Great Britain. No one would venture to dictate to the principal Metropolitan and provincial hospitals, or to suggest in any way that their examinations are not quite up to date, and perfectly conducted. What we do want, is that these hospitals, which themselves are *sans peur et sans reproche*, should take a wider view of the profession generally—that they should come down from their proud isolation and recognise all the evils quoted in that admirable article on training, and realise their responsibility with regard to the smaller public hospitals, and the still greater evil, private nursing homes. The two largest training schools in Melbourne set the example of at once taking advantage of the services of the Central Board of Examiners for their pupils; the other hospitals followed the lead. As a matter of course, the benefit was not to those two hospitals, but to the smaller ones, which could not very well refuse when the Melbourne and the Alfred expressed their willingness for the Board to examine their probationers. As to the English private nursing homes, the best of these would not dream of employing probationers for patients who paid from £3 3s. to £6 6s. per week, but in the inferior ones, unfortunately, the girls are encouraged by promises of training, indifferently kept, to work for very small remuneration, and sometimes even payment of a fee. They are usually told that the training would be equivalent to that of a public hospital, that Dr. So-and-so would give a certificate, and keep the nurse in work. The medical profession has not been quite blameless in this matter, for the doctors forget that they make themselves responsible for the false position in which the nurse is placed. Nor must the hospital matron be quite exempt from censure. The suggestion that a nurse trained in a small hospital should have her second or third year in a large one, admirable as it is, will meet with great opposition from the heads of training schools, and yet it appears the only way out of the difficulty. Children's hospitals should certainly be regarded as special training schools. State registration will make a requisite training compulsory. After the usual period of grace allowed to those who had vested rights, no special nurse should be accepted who has not previously had three years' general training. If the large hospitals will enter into some arrangement with the small ones and give the probationers of the latter one or two years (to be included in the three years' course), a great benefit will be conferred both on nurses and the struggling institutions, which would, if they could, desist from training probationers and employ only trained nurses. As to the arguments against registration, when we come across a nurse who has had three years' training and still is ignorant, we may well feel aghast at what she would have been with less. On the contrary, when we find a really capable nurse, who was trained in some irregular way for a short period, we regret she had not the opportunity of making herself really proficient. State registration can in no way detract from the strong, reliant, tender, sympathetic woman, the ideal nurse, but it will be proof that she has added to her strength wisdom, to her sympathy knowledge, and to her reliance skill.

### TRAVEL NOTES AND QUERIES.

By our Travel Correspondent.

ROUEN IN JANUARY (Policy 609).—I should not advise Rouen at this time of the year; it is cold and somewhat damp in the winter and unless you are artistic and have a marked interest in architecture—domestic and ecclesiastical—you would, I think, find it very dull, all excursions up and down the Seine, etc., being impracticable. Dieppe is also out of the question being extremely expensive. I would suggest as more suitable St. Servan, in the Bay of St. Malo, reached directly from Southampton. Return fare, second class, £1 19s. If you send a reply prepaid foreign letter-card to Madame Pallot, Maison Mathias, St. Servan, France, and ask for her lowest terms for the month of January, she will reply, and if you can get there you will be extremely comfortable and not dull. The terms will be from 5 to 6 francs per day. For a month I think she might take you at this season for 25s. per week. You will be happy there and being close to Dinard it is lively. Dinard itself is one of the most expensive places on the Continent. Please note that nurses' homes in the sense you mean do not exist abroad. If you cannot afford from 25s. to 30s. and do not object to Belgium, I could tell you of something still cheaper, but it would be in a convent.

RULES IN REGARD TO CORRESPONDENCE FOR THIS SECTION.—All questioners must use a pseudonym for publication, but the communication must also bear the writer's own name and address as well, which will be regarded as confidential. All such communications to be addressed "Travel Correspondent, 'Nursing Section of *The Hospital*,' 28 Southampton Street, Strand." No charge will be made for inserting and answering questions in the inquiry column, and all will be answered in rotation as space permits. If an answer by letter is required, a stamped and addressed envelope must be enclosed, together with 2s. 6d., which fee will be devoted to the objects of "The Hospital" Convalescent Fund. Ten days must be allowed before an answer can be published.

### A Book and its Story.

THE MISADVENTURES OF A CANON.*

When an author or the writer of a play chooses a clergyman as the peg upon which to hang incidents necessary to the development of the plot, the position is invariably one in which the clergyman is exposed to ridicule. The satire, good-humoured enough in most cases, and having its origin in the deep-seated prejudice existing in the British mind against anything that savours of cant and hypocrisy—two failings erroneously associated in the minds of many men with the prefix "Reverend," shows also a tendency to ignore the best type of English clergyman. The object of "The Canon in Residence," written from within the clerical fold, is apparently to illustrate, by means of an improbable but very amusing story, the proverb of "O that some fay the gift wad' gie us, to see ourselves as others see us."

The hero, the Reverend John Smith, conceives the idea that a few weeks in Switzerland would be beneficial from two points of view. He had a book on hand which, before it was completed, required certain information only to be found in works which were in the library at Zurich, and the weeks intervening before Lent were favourable ones in which to take a little recreation. So, without further premeditation, he starts on the journey which, through meeting with a voluble individual in a check suit, sets a train of adventures moving which involve the hapless canon in a network of intrigue. There is nothing tragic in his description. "The Reverend John Smith was a middle-aged man of medium height, dressed very correctly as an English clergyman. His hair was just a little tinged with grey, as were also his side whiskers. The rest of his face was clean-shaven and of rather an ecclesiastic type, but there was that half-apparent upward turn of the corners of his mouth that told he was by no means devoid of humour, while his eyes were distinctly of a kindly type." It was really his literary hobby that had brought Mr. Smith to Switzerland, and after he had despatched the business of research at Zurich, he had yet some ten days to spare, which he decided to spend at St Moritz. Having arrived at Thusis, he got out of the little narrow gauge train, and giving his bag into the hands of the hotel porter he strolled up the steep ascent that leads from the station to the Hôtel des Postes. At this particular time of the year the hotels of Thusis are practically empty, but a few passing travellers stay the night there. Mr. Smith found himself the only one bound for the Hôtel des Postes. But upon entering the smoking-room he discovered one other solitary person. "He was a man about the same height and build as the clergyman, but of a very different type. He wore a heavy dark moustache, and was in all respects the sort of man that at a glance one would put down as a typical British tourist of a class to be met with all over the Continent. One cannot go up the Rhine on a summer's day, one cannot take a trip on the Italian Lakes in the spring, without meeting such men similarly dressed. . . . The stranger looked up at the entry of the clergyman, taking stock of him with eyes that were sharp and alert. He surveyed him narrowly from head to foot with a restless, apprehensive expression." The stranger listened to Mr. Smith's inquiry and smiled as he addressed the host and asked for a bed. "It was made in a tone that no other profession ever adopts. An expert in human nature can sit with his back to an hotel entrance when a host of tourists come rushing in from an incoming train, and he will pick out the English parson abroad nine times out of ten by the simple intonation of his voice as he asks for a bed. Perhaps it reminds one of the Litany monotoned." Mr. Smith was taken at once to a room, No. IX., which he was assured was the best in the house. This was not the case, for room No. X. had already been given to the stranger in the hall. "Both rooms were warmed by the same stove, and mine host charged each guest for the warming." After dinner he and the stranger entered into conversation. He found him well informed and able to tell him many things about St. Moritz and the winter there. Then the conversation took a slightly ecclesiastical turn. Something was said about the clergy, and the stranger declared that as a class they knew very little about human nature; that between the clergy and the laity a great gulf was fixed; and that the one great barrier to a better understanding was the clerical uniform. Men were not natural in the presence of it. "By virtue of your office and your uniform—that collar, for instance—you create, shall we say, a halo around you? That's the gulf." Mr. Smith listened to this and other plain-spoken suggestions from the stranger, and at last remarked, "You say we clergy do not understand men. Perhaps you can tell me how to do it?" The stranger leaned forward and touched the other's coat. "Take off this," he said, "and that," he went on, pointing to Mr. Smith's collar. "Put on ordinary clothes and drop the parson. Then go and mix with men, and you'll see I'm right." Mr. Smith replied that surely he would not have him go about his parish of Market Shapborough dressed, for instance, as he was, and his eyes fell on the loud check suit. "You are going to spend a week at St. Moritz just at the height of the winter season. This will be a glorious opportunity for you to become a layman for the time being. You'll meet all sorts there—snobs, scientists, opinions of the brainy, and opinions of the brainless. It's a little world in itself. You lose your chance of making the best of it by going there as a parson." The stranger and Mr. Smith had adjoining bedrooms. He retired early, leaving Mr. Smith to meditate upon his suggestions. Before saying good night he had handed an English paper to him. Glancing at the ecclesiastical intelligence column, his eye was arrested by his own name. He saw that the vacant canonry of Frattenburg Cathedral had been offered to him. "Canon Smith!" For a few minutes he revelled in the thought silently; and who would not forgive him? . . . The canonry was worth £500 a year—riches to him. The smoke which he puffed upward seemed to take the form of a white-robed figure preceded by a solemn verger, carrying a silver wand, marching to his stall in Frattenburg Cathedral." He is aroused from his vision by the stranger's voice offering him a cigar in place of his own that had gone out. Mr. Smith takes the last one left in the case. "Dear me, but it's your last." "I've plenty more upstairs," said the stranger, "I'll go and get some." The next minute he was on the first floor landing. As is customary in Continental hotels, there was a double door leading from No. IX. to No. X. Each door had its own key on the inside. The stranger entered Mr. Smith's room, turned on the electric light, unlocked the door, and put the key into his pocket. He could not enter the room from his own. When Mr. Smith awakes the following morning he finds the stranger has made off with his clerical clothes and left his own in their place! Driven to desperation at the discovery, Mr. Smith has yet no time to waste. He must go on, and he must dress. "He put on the black evening dress trousers, struggled into the least gaudy of the ties, and then clothed himself with the terrible coat and waistcoat." In this motley garb he starts for St. Moritz. His adventures there and the difficulties arising from them when he returns home are graphically set forth in "The Canon in Residence."

* "The Canon in Residence." By Victor L. Whitechurch. T. Fisher Unwin. 6s.

### Echoes from the Outside World.

### Christmas at Sandringham

The King and Queen spent Christmas at Sandringham in the usual quiet manner. On Christmas Eve the Royal Family met in the large ball-room, where a fir tree had been installed as the Christmas tree, and on it were hung presents intended for members of the Royal Family and the Household. On Sunday morning the King and Queen, the Prince and Princess of Wales, Princess Victoria, Princess Louise, Duchess of Fife, and Princess Charles of Denmark, attended Divine service in Sandringham Church. The service was conducted by Canon Hervey, and there was no sermon. All the members of the Royal Family received Holy Communion. On Saturday afternoon the workmen in the King's employ and the cottagers on the Sandringham estate assembled at Sandringham in order to receive substantial joints of beef, which his Majesty distributes at Christmastide. Both the King and the Prince of Wales were present at the distribution, which took place in the Royal coach house. On Monday they enjoyed several hours' shooting, the Queen and the Princesses taking lunch with them, and remaining, despite the damp weather, until the shooting ceased at half-past three. The Queen sent a number of presents to children on the Sandringham estate.

### Decree by the Tsar of Russia.

On Monday the Tsar's Imperial decree to the Senate under the title of "A Scheme for the Improvement of the Administration of the State" was issued. The reforms advocated by his Majesty are chiefly, peasant legislation on the basis of an extension of powers of the local committees recently nominated by the Government; increased powers for town councils and other elective bodies; the introduction, besides Government District Zemstvos, of parish Zemstvos in strict unity therewith; the introduction of greater unity in criminal and civil law institutions of the Empire; the State insurance of workmen; the revision of the special laws, introduced owing to the disturbed condition of the country; the revision of the laws concerning sects and other religious committees; and the revision of the Press laws. The Tsar expresses his confidence that the proposed reforms will afford satisfaction to all good subjects of Russia.

### The Siege of Port Arthur.

A Tokio telegram says that the Japanese besieging army occupied Taliuchiatum on Saturday, and officially announces the fall of the whole of the Russian advanced positions in front of the Japanese right. . . . The Emperor of Japan has issued an Imperial Rescript addressed to Admiral Togo, in which he observes, "We hear with great satisfaction that our torpedo flotillas engaged in the work required of them at Port Arthur have gallantly and successfully accomplished the duties they were called upon to perform and in so doing have had to brave the dangers of storms and shells by day and night, and that notwithstanding difficulties they have succeeded in discharging their duties without the least confusion, rendering mutual assistance. We especially note their brave and loyal performance of the duties required of them, and express our approbation of their gallant behaviour." . . . General Kuropatkin, in his despatches of December 24th and 25th mentions some slight Russian successes. . . . A telegram despatched from Tokio on Christmas Day states that thousands of recruits and Reservists are assembled in the capital, and that everywhere the new troops are drilling and receiving equipment. It is intended to bring up Marshal Oyama's command to 500,000 men, besides heavily reinforcing the Artillery at his disposal. Extensive preparations are also being made for the defence of Formosa in the southern islands, in anticipation of the Baltic Fleet attempting to seize a base among them.

### English Passengers in a French Railway Accident.

In spite of the fog, the only notable railway accident which occurred in England during the Christmas holidays was near Aylesbury, on the Great Central main line. A newspaper train was derailed and four men were killed. But in France on Friday night there was a very serious collision at Porte de la Chapelle, a few minutes' distance from the Gare du Nord, involving the loss of more than a dozen lives and injuries to about 40 persons. The sufferers were confined to the passengers in the express from Lille, those in the Boulogne train, which included a large number of English people, being practically unhurt. The accident appears to have primarily been due to the dense fog which hung over Paris and the suburbs, rendering the signals difficult, if not impossible, to distinguish. In describing the remarkable immunity of the travellers in the train from Boulogne, a New York medical man says "there was no panic, no unseemly excitement. Gendarmes and civilian passengers, train-hands and soldiers on furlough, worked together in extricating the injured." One of the badly injured was a medical man, who, after being for over three hours pinned beneath a mass of wreckage, said he would wait as long as necessary, and was only concerned that someone should go and tell his wife not to be alarmed. Subsequently, at the Paris Morgue, Professor Berthelot was led away speechless on seeing among the dead a soldier-grandson, whose homecoming was to make Christmas more than usually joyful.

### Death of a Famous Aeronaut.

The Rev. J. M. Bacon died at his residence, Sunnyside, Coldash, near Newbury, on Christmas Day. Mr. Bacon, who was 58 years old, was a famous aeronaut, and he had not taken regular clerical duty for a long time. His life was, in fact, devoted to scientific research, and his intrepidity as a balloonist was shown on several occasions. He took part in the eclipse expeditions of the British Astronomical Association to Vadso, Lapland, in 1896; to India in 1898, when he was in charge of the expedition; and to Wadesborough, North Carolina, in 1900, when he was also in charge. On one occasion he ascended as far as two miles above the earth. His discoveries by means of sounding a horn at frequent intervals were of much interest.

### London's New Place of Entertainment.

On Christmas Eve the Coliseum in St. Martin's Lane was opened to the public. There are four performances a day, each of two hours' duration; one of which commences at twelve and is repeated at six, whilst the other takes place at three and also at nine. The hall itself is exceedingly large, the great dome appearing almost as lofty as that of St. Paul's, and it is very handsome and well appointed. A striking innovation is that every seat in the house, ranging from 6d. up to 4s., can be booked in advance. Besides the performers on the stage, there is a choir of over fifty voices. Its members are grouped on either side of the stage in two richly-furnished and marble-framed alcoves, which flank the proscenium, and are arrayed in costumes of spotless white. Beautiful scenery is an important feature of the show, and all the songs, the feats of juggling, and the comic interludes have their own scenic setting; love songs are rendered on the banks of the Ganges, a charming Irish ditty is sung from an Irish cottage, and a witch's quick-change scene is in the Catskill Mountains. "The Last Load," depicting rural life in the early Victorian period, and the race course on Derby Day, where real horses run real races, are noteworthy items in a wonderfully varied programme.

### Notes and Queries.

**REGULATIONS.**

**The Editor is always willing to answer in this column, without any fee, all reasonable questions, as soon as possible.**

**But the following rules must be carefully observed :—**

1. **Every communication must be accompanied by the name and address of the writer.**
2. **The question must always bear upon nursing, directly or indirectly.**

**If an answer is required by letter a fee of half-a-crown must be enclosed with the note containing the inquiry.**

*Canada.*

(96) Can you tell me the best place to write to for information about work as maternity nurse in Canada, either privately, or in connection with a home or hospital?—*Nurse Nettie.*

Write to the Lady Superintendent, the Victorian Order of Nurses for Canada, Headquarters, 578 Somerset Street, Ottawa, Ontario, Canada.

*Plague Nursing.*

(97) I want to go out to India as a plague nurse, but I do not wish to train in general nursing, as I do not like surgical work. Would three years' training and certificate from a large fever hospital be sufficient?—*Anxious.*

A certificate for three years' training in general nursing is essential for every appointment of importance, but in times when the demand is great your services might be accepted.

*Grains, Minims.*

(98) How many grains are there in a minim? Morphia is some times administered in one-third grain doses. How much is one-third grain?—*A Nurse.*

This elementary information can be found in any text-book on nursing; but we are sorry to see the question asked in connection with such a dangerous drug as morphia.

*Asylums in South Africa.*

(99) Will you please tell me the names of some asylums near Durban or Capetown?—*Nurse R.*

Vankenberg Hospital for the Insane, Mowbray, Capetown. Other asylums in Cape Colony are Port Alfred Asylum and Fort Beaufort Asylum. There are also in Grahamstown the Grahamstown Asylum, the Chronic Sick Hospital, and the Institute for Imbecile Children. There is a large Government Asylum for the Insane in Maritzburg, but none in Durban.

*Nurses for India.*

(100) Please tell me where I can obtain further information about the nurses who are wanted for India?—*E. H. G.*

I am very anxious to go out to India, and I should be very grateful if you would kindly help me in the matter?—*H. McK.*

Applications may be sent to Miss Mackenzie, the Secretary of the Women's Work Committee of the Society for the Propagation of the Gospel, 19 Delahay Street, Westminster, S.W.; but, as we stated in our issue of last week, there are openings only for nurse-missionaries.

*Homes for Epilepsy and Annuities.*

(101) Will you kindly give me (1) the addresses of any Homes for Epilepsy near London, and (2) tell me if there are any annuities for poor widows of 6s. per week after 60 years of age?—*H. B.*

(1) You do not mention if you want homes for male or female patients. (2) Possibly you allude to the Widows' Friend Society, 57A Coleman Street, E.C., which gives pensions of £6 a year. For further information consult "Burdett's Hospitals and Charities."

*Knitting.*

(102) Referring to Query 85, The Secretary of the British and Foreign Bible Association, 206 Great Portland Street, W., writes that "he should be only too glad to receive orders for knitting, etc., and will see that they are placed in the hands of deserving and efficient blind people."

**Handbooks for Nurses.**

| | Post Free. |
|---|---|
| How to Become a Nurse: "The Nursing Profession" ... | 2s. 4d. |
| "Nurses' Pronouncing Dictionary." Terms and Treatment... ... ... ... ... ... ... | 2s. 0d. |
| "A Handbook for Nurses." Dr J. K. Watson ... ... | 5s. 4d. |
| "Elementary Physiology for Nurses" ... ... ... | 2s. 0d. |
| "Swedish Massage and Movements" ... ... ... ... | 2s. 6d. |

**Of all booksellers or of The Scientific Press, Limited, 28 & 29 Southampton Street, Strand, London, W.C.**

### For Reading to the Sick.

BEYOND THE YEARS.

Beyond the years the prayer for rest
Shall beat no more within the breast;
The darkness clears,
And Morn perched on the mountain's crest
Her form uprears—
The day that is to come is best,
Beyond the years.

Beyond the years the soul shall find
That endless peace for which it pined,
For light appears,
And to the eyes that still were blind
With blood and tears,
Their sight shall come all unconfined—
Beyond the years.

*Paul L. Dunbar.*

Prayer.

O Thou, who art ever the same, give us faith to bear with patience all that Thou, in Thy loving wisdom may see fit to send us, in the year upon which we are entering. Give us grace to realise that this transitory life is but a probation for an eternal, and happier one, to which in Thy mercy bring us. Amen.

The deliverance of the soul from all useless and selfish and unquiet cares, brings to it an unspeakable peace and freedom; this is true simplicity. This state of entire resignation and perpetual acquiescence produces true liberty; and this liberty brings perfect simplicity. The soul which knows no self-seeking, no interested ends, is thoroughly candid; it goes straight forward without hindrance; its path opens daily more and more to "perfect day," in proportion as its self-renunciation and its self-forgetfulness increase: and its peace, amid whatever troubles beset it, will be as boundless as the depths of the sea.—*Fenelon.*

The way of suffering is the witness which a soul bears to itself.

Suffering was a curse from which man fled; now it becomes a purification of the soul, a sacred trial sent by eternal Love, a divine dispensation meant to sanctify and ennoble us, an acceptable aid to faith, a strange initiation into happiness. O power of belief! All remains the same yet all is changed. A new certitude arises to deny the apparent and the tangible; it pierces through the mystery of things, it places an invisible Father behind visible Nature, it shows us joy shining through tears, and makes of pain the beginning of joy.—*Amiel.*

Every contradiction of our will, every little ailment, every petty disappointment, will, if we take it patiently, become a blessing. So, walking on earth, we may be in heaven. . . . Ill-health, the daily accidents with which God has mercifully strewed our paths, instead of ruffling or disturbing our peace, may cause His peace to be shed abroad in our hearts abundantly.—*E. B. Pusey.*

The purer life draws nigher
And earth's morning star climbs higher
Every year.
And earth's hold on us grows slighter
And the heavy burdens lighter
Every year.

*Anon.*

